# Supplementary material for: In vivo transition in chromatin accessibility during differentiation of deep-layer excitatory neurons in the neocortex
Source: Development. 2025 Jun 27;152(13):dev204564. doi: 10.1242/dev.204564 (PMC12268177; doi:10.1242/dev.204564)
Supplement: Supplementary information [file develop-152-204564-s1.pdf]

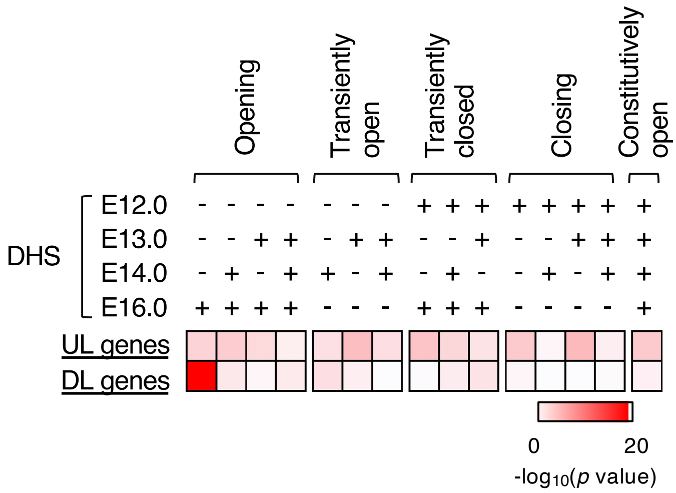

Fig. S1. Enrichment of deep layer-specific genes in the opening genes.
Significance of the overlap between upper layer- or deep layer-specific genes and genes with (+) or without (-) DHSs at their promoter regions was determined using the Fisher's exact test.

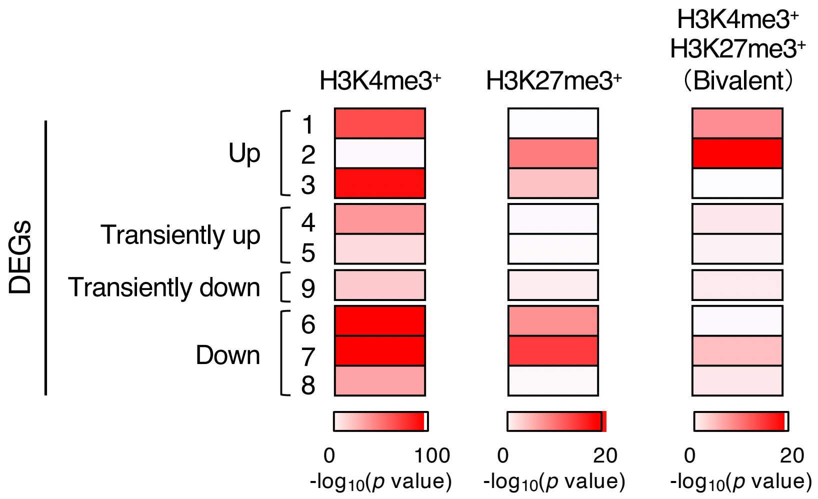

Fig. S2. Enrichment of bivalent genes in the upregulated DEGs.
Significance of the overlap between DEG clusters determined in Fig. 2 and H3K4me3$^+$ H3K27me3$^-$, H3K4me3$^-$ H3K27me3$^+$, and H3K4me3$^+$ H3K27me3$^+$ genes was determined using the Fisher's exact test.

Development • Supplementary information

Table S1. Gene list with the summary of transcriptomic, chromatin, and epigenetic analysis in this study, related to Fig. 2–7.

Columns A–G: Information on the genes analyzed in this study.

Column H–S: Results of RNA-seq during neuronal differentiation.

Column T: DEG cluster numbers.

Column U–AB: DHS-positive genes at their promoters (U–X) or enhancers (Y–AB). Numbers in the enhancer column indicate the number of DHSs annotated in the enhancer regions.

Columns AC–AF: Results of RNA-seq of the upper or deep layers in Belgard et al., 2011.

Column AG-AH: Upper layer- or deep-layer-specific genes indicated by Belgard et al., 2011

Column AI: Genes upregulated or downregulated by BDNF treatment.

Column AJ–AK: Promoter regions of H3K4me3^{+} and H3K27me3^{+} genes.

Column AL–AM: Promoter regions of Dmrt3^{+} or Dmrta2^{+} genes were determined using the data from Konno et al. (2019).

Columns AN–AO: Results of RNA-seq of *Dmrt3* and *Dmrta2* double knockouts in Konno et al., 2019.

Column AP–AQ: Genes upregulated or downregulated by the *Dmrt3* and *Dmrta2* double knockout in Konno et al., 2019.

Column AR–AY: Results of RNA-seq of *Dmrt3* and *Dmrta2* double knockouts from Desmaris et al., 2018.

Column AZ–BA: Genes up- or downregulated by *Dmrt3* and *Dmrta2* double knockout in Desmaris et al., 2018.

Available for download at
https://journals.biologists.com/dev/article-lookup/doi/10.1242/dev.204564#supplementary-data

Table S2. GO analysis (biological process and cellular components) for genes with DHSs at each stage, related to Fig. 4.

Available for download at
https://journals.biologists.com/dev/article-lookup/doi/10.1242/dev.204564#supplementary-data

Table S3. HOMER motif analysis (known motif) for DHSs at each stage, related to Fig. 4.

Available for download at
https://journals.biologists.com/dev/article-lookup/doi/10.1242/dev.204564#supplementary-data

Development • Supplementary information

Table S4. HOMER motif analysis (de novo motif) for all DHSs at each stage, related to Fig. 4.

Available for download at
https://journals.biologists.com/dev/article-lookup/doi/10.1242/dev.204564#supplementary-data

Table S5. HOMER motif analysis (de novo motif) for specific DHSs at each stage, related to Fig. 4.

Available for download at
https://journals.biologists.com/dev/article-lookup/doi/10.1242/dev.204564#supplementary-data

Table S6. ChIP-Atlas analysis for DHSs at each stage, related to Fig 4.

Available for download at
https://journals.biologists.com/dev/article-lookup/doi/10.1242/dev.204564#supplementary-data

Table S7. Microarray analysis of primary neuronal culture with or without BDNF treatment, related to Figu. 5.

Available for download at
https://journals.biologists.com/dev/article-lookup/doi/10.1242/dev.204564#supplementary-data

Table S8. Changes in the expression of genes encoding components of PcG or TrxG. A summary of the expression levels of genes encoding PcG or TrxG components during neuronal differentiation from our RNA-seq dataset (Data S1) is shown. A list of the components of PcG and TrxG (Schuettengruber et al., 2017) used. As sex chromosomes were excluded from our analysis (as described in the Materials and methods) there were no data for genes on chromosome X.

Available for download at
https://journals.biologists.com/dev/article-lookup/doi/10.1242/dev.204564#supplementary-data
