# Supplementary material for: In vivo transition in chromatin accessibility during differentiation of deep-layer excitatory neurons in the neocortex
Source: Development. 2025 Jun 27;152(13):dev204564. doi: 10.1242/dev.204564 (PMC12268177; doi:10.1242/dev.204564)
Supplement: Table S4. [file develop-152-204564-TableS4.zip › TableS4.pdf]

Homer *de novo* Motif Results (E12_motif_bg_random/)

Known Motif Enrichment Results
Gene Ontology Enrichment Results
If Homer is having trouble matching a motif to a known motif, try copy/pasting the matrix file into STAMP
More information on motif finding results: HOMER | Description of Results | Tips
Total target sequences = 22083
Total background sequences = 30000
* - possible false positive

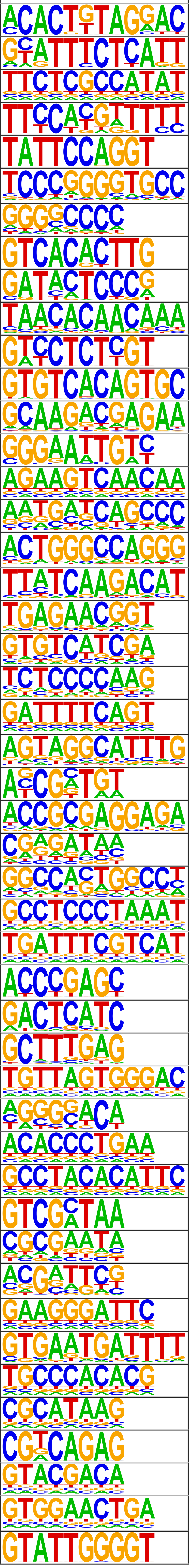

Rank	Motif	P-value	log P-pvalue	% of Targets	% of Background	STD(Bg STD)	Best Match/Details	Motif File
1		1e-54	-1.260e+02	0.19%	0.01%	498.4bp (19.6bp)	OSR1/MA1542.1/Jaspar(0.614) More Information | Similar Motifs Found	motif file (matrix)
2		1e-50	-1.152e+02	0.21%	0.01%	453.9bp (36.0bp)	POU6F1(var.2)/MA1549.1/Jaspar(0.647) More Information | Similar Motifs Found	motif file (matrix)
3		1e-47	-1.102e+02	0.17%	0.00%	464.2bp (0.0bp)	E2F8/MA0865.1/Jaspar(0.693) More Information | Similar Motifs Found	motif file (matrix)
4		1e-46	-1.064e+02	0.17%	0.01%	465.2bp (55.1bp)	Prdm15/MA1616.1/Jaspar(0.773) More Information | Similar Motifs Found	motif file (matrix)
5		1e-37	-8.533e+01	0.17%	0.01%	449.9bp (29.1bp)	TEAD2(TEA)/Py2T-Tead2-ChIP-Seq(GSE55709)/Homer(0.727) More Information | Similar Motifs Found	motif file (matrix)
6		1e-27	-6.306e+01	2.95%	1.86%	287.6bp (46.6bp)	ZNF143|STAF(Zf)/CUTLL-ZNF143-ChIP-Seq(GSE29600)/Homer(0.633) More Information | Similar Motifs Found	motif file (matrix)
7		1e-25	-5.900e+01	12.07%	9.86%	257.3bp (49.2bp)	PLAGL2/MA1548.1/Jaspar(0.919) More Information | Similar Motifs Found	motif file (matrix)
8		1e-24	-5.595e+01	0.18%	0.02%	539.2bp (10.9bp)	TBX1/MA0805.1/Jaspar(0.758) More Information | Similar Motifs Found	motif file (matrix)
9		1e-23	-5.314e+01	0.22%	0.03%	246.7bp (40.4bp)	DPRX/MA1480.1/Jaspar(0.766) More Information | Similar Motifs Found	motif file (matrix)
10		1e-21	-4.900e+01	0.12%	0.01%	531.3bp (19.2bp)	PB0121.1_Foxj3_2/Jaspar(0.773) More Information | Similar Motifs Found	motif file (matrix)
11		1e-21	-4.893e+01	0.15%	0.02%	706.6bp (36.9bp)	PB0139.1_Irf5_2/Jaspar(0.627) More Information | Similar Motifs Found	motif file (matrix)
12		1e-20	-4.620e+01	0.11%	0.01%	297.2bp (25.0bp)	MEIS1/MA0498.2/Jaspar(0.600) More Information | Similar Motifs Found	motif file (matrix)
13		1e-20	-4.620e+01	0.11%	0.01%	303.5bp (24.6bp)	PB0138.1_Irf4_2/Jaspar(0.572) More Information | Similar Motifs Found	motif file (matrix)
14		1e-19	-4.469e+01	0.15%	0.02%	239.3bp (44.9bp)	GFY(?)/Promoter/Homer(0.766) More Information | Similar Motifs Found	motif file (matrix)
15		1e-19	-4.450e+01	0.12%	0.01%	454.3bp (22.2bp)	Nr2e1/MA0676.1/Jaspar(0.777) More Information | Similar Motifs Found	motif file (matrix)
16		1e-18	-4.343e+01	0.11%	0.01%	378.1bp (28.7bp)	ZNF341/MA1655.1/Jaspar(0.670) More Information | Similar Motifs Found	motif file (matrix)
17		1e-18	-4.301e+01	5.48%	4.21%	333.0bp (49.5bp)	ZNF416(Zf)/HEK293-ZNF416.GFP-ChIP-Seq(GSE58341)/Homer(0.666) More Information | Similar Motifs Found	motif file (matrix)
18		1e-18	-4.203e+01	0.12%	0.01%	855.7bp (33.1bp)	Smad4/MA1153.1/Jaspar(0.665) More Information | Similar Motifs Found	motif file (matrix)
19		1e-16	-3.871e+01	4.44%	3.35%	442.0bp (48.2bp)	PB0137.1_Irf3_2/Jaspar(0.695) More Information | Similar Motifs Found	motif file (matrix)
20		1e-16	-3.753e+01	7.43%	6.04%	425.5bp (48.8bp)	MEIS1/MA0498.2/Jaspar(0.711) More Information | Similar Motifs Found	motif file (matrix)
21		1e-15	-3.660e+01	7.15%	5.80%	372.8bp (47.6bp)	Znf281/MA1630.1/Jaspar(0.671) More Information | Similar Motifs Found	motif file (matrix)
22		1e-15	-3.541e+01	0.09%	0.01%	476.3bp (20.6bp)	Hoxd12(Homeobox)/ChickenMSG-Hoxd12.Flag-ChIP-Seq(GSE86088)/Homer(0.649) More Information | Similar Motifs Found	motif file (matrix)
23		1e-15	-3.541e+01	0.09%	0.01%	133.0bp (33.6bp)	Hic1/MA0739.1/Jaspar(0.647) More Information | Similar Motifs Found	motif file (matrix)
24		1e-15	-3.537e+01	6.80%	5.51%	330.3bp (44.4bp)	CUX2/MA0755.1/Jaspar(0.674) More Information | Similar Motifs Found	motif file (matrix)
25		1e-15	-3.528e+01	0.12%	0.01%	91.2bp (42.9bp)	ZNF263/MA0528.2/Jaspar(0.647) More Information | Similar Motifs Found	motif file (matrix)
26		1e-14	-3.388e+01	7.87%	6.52%	535.0bp (45.9bp)	GATA3(Zf)/iTreg-Gata3-ChIP-Seq(GSE20898)/Homer(0.708) More Information | Similar Motifs Found	motif file (matrix)
27		1e-14	-3.283e+01	0.09%	0.01%	157.1bp (15.6bp)	FXR(NR),IR1/Liver-FXR-ChIP-Seq(Chong_et_al.)/Homer(0.775) More Information | Similar Motifs Found	motif file (matrix)
28		1e-14	-3.283e+01	0.09%	0.01%	409.2bp (70.2bp)	PB0092.1_Zbtb7b_1/Jaspar(0.665) More Information | Similar Motifs Found	motif file (matrix)
29		1e-14	-3.283e+01	0.09%	0.01%	495.0bp (5.4bp)	PH0037.1_Hdx/Jaspar(0.763) More Information | Similar Motifs Found	motif file (matrix)
30		1e-14	-3.241e+01	2.89%	2.10%	311.6bp (45.6bp)	ZNF519(Zf)/HEK293-ZNF519.GFP-ChIP-Seq(GSE58341)/Homer(0.664) More Information | Similar Motifs Found	motif file (matrix)
31		1e-13	-3.142e+01	4.74%	3.72%	317.7bp (48.3bp)	NFE2/MA0841.1/Jaspar(0.818) More Information | Similar Motifs Found	motif file (matrix)
32		1e-13	-3.111e+01	7.84%	6.54%	485.2bp (47.5bp)	TCF7/MA0769.2/Jaspar(0.767) More Information | Similar Motifs Found	motif file (matrix)
33		1e-13	-3.030e+01	0.08%	0.01%	85.1bp (25.9bp)	NR1D2/MA1532.1/Jaspar(0.693) More Information | Similar Motifs Found	motif file (matrix)
34		1e-12	-2.854e+01	11.90%	10.38%	394.4bp (49.5bp)	AR-halfsite(NR)/LNCaP-AR-ChIP-Seq(GSE27824)/Homer(0.767) More Information | Similar Motifs Found	motif file (matrix)
35		1e-12	-2.782e+01	0.08%	0.01%	112.7bp (22.1bp)	ZSCAN4/MA1155.1/Jaspar(0.771) More Information | Similar Motifs Found	motif file (matrix)
36		1e-12	-2.782e+01	0.08%	0.01%	82.8bp (33.2bp)	TEAD2/MA1121.1/Jaspar(0.661) More Information | Similar Motifs Found	motif file (matrix)
37 *		1e-10	-2.454e+01	0.12%	0.03%	96.1bp (32.2bp)	PB0179.1_Sp100_2/Jaspar(0.715) More Information | Similar Motifs Found	motif file (matrix)
38 *		1e-10	-2.433e+01	2.19%	1.60%	228.1bp (42.9bp)	PB0138.1_Irf4_2/Jaspar(0.696) More Information | Similar Motifs Found	motif file (matrix)
39 *		1e-10	-2.376e+01	4.95%	4.05%	283.9bp (45.8bp)	Npas4(bHLH)/Neuron-Npas4-ChIP-Seq(GSE127793)/Homer(0.627) More Information | Similar Motifs Found	motif file (matrix)
40 *		1e-10	-2.304e+01	0.07%	0.01%	202.6bp (9.6bp)	PH0129.1_Otx1/Jaspar(0.678) More Information | Similar Motifs Found	motif file (matrix)
41 *		1e-10	-2.304e+01	0.07%	0.01%	444.6bp (43.6bp)	PH0037.1_Hdx/Jaspar(0.679) More Information | Similar Motifs Found	motif file (matrix)
42 *		1e-9	-2.164e+01	0.08%	0.01%	96.1bp (30.2bp)	HIC2/MA0738.1/Jaspar(0.727) More Information | Similar Motifs Found	motif file (matrix)
43 *		1e-9	-2.164e+01	0.08%	0.01%	74.0bp (37.9bp)	HLF/MA0043.3/Jaspar(0.639) More Information | Similar Motifs Found	motif file (matrix)
44 *		1e-8	-2.039e+01	1.51%	1.07%	432.1bp (45.3bp)	MF0002.1_bZIP_CREB/G-box-like_subclass/Jaspar(0.686) More Information | Similar Motifs Found	motif file (matrix)
45 *		1e-7	-1.757e+01	0.09%	0.02%	90.2bp (46.6bp)	PB0094.1_Zfp128_1/Jaspar(0.769) More Information | Similar Motifs Found	motif file (matrix)
46 *		1e-7	-1.746e+01	0.08%	0.01%	61.9bp (8.8bp)	ZNF189(Zf)/HEK293-ZNF189.GFP-ChIP-Seq(GSE58341)/Homer(0.752) More Information | Similar Motifs Found	motif file (matrix)
47 *		1e-6	-1.428e+01	0.05%	0.01%	53.7bp (30.7bp)	RUNX3/MA0684.2/Jaspar(0.665) More Information | Similar Motifs Found	motif file (matrix)

Homer *de novo* Motif Results (E13_motif_bg_random/)

[Known Motif Enrichment Results](#)
[Gene Ontology Enrichment Results](#)
If Homer is having trouble matching a motif to a known motif, try copy/pasting the matrix file into [STAMP](#)
More information on motif finding results: [HOMER](#) | [Description of Results](#) | [Tips](#)
Total target sequences = 20370
Total background sequences = 29000
* - possible false positive

Rank	Motif	P-value	log P-pvalue	% of Targets	% of Background	STD(Bg STD)	Best Match/Details	Motif File
1	TTTCTCGCCATA	1e-71	-1.645e+02	0.24%	0.01%	547.9bp (24.3bp)	E2F8/MA0865.1/Jaspar(0.741) More Information | Similar Motifs Found	motif file (matrix)
2	TTTTTCAGCG	1e-58	-1.348e+02	0.25%	0.01%	514.7bp (21.5bp)	Rhox11/MA0629.1/Jaspar(0.664) More Information | Similar Motifs Found	motif file (matrix)
3	TTTTCCATGATT	1e-55	-1.283e+02	0.27%	0.01%	468.2bp (12.3bp)	NFAT5/MA0606.1/Jaspar(0.728) More Information | Similar Motifs Found	motif file (matrix)
4	TGTGAATTTCTC	1e-55	-1.276e+02	0.24%	0.01%	444.3bp (25.5bp)	Foxh1(Forkhead)/hESC-FOXH1-ChIP-Seq(GSE29422)/Homer(0.643) More Information | Similar Motifs Found	motif file (matrix)
5	CCGTATTTCA	1e-51	-1.186e+02	0.19%	0.01%	439.5bp (23.3bp)	PB0106.1_Arid5a_2/Jaspar(0.698) More Information | Similar Motifs Found	motif file (matrix)
6	TTTCTCATTT	1e-45	-1.048e+02	0.27%	0.02%	447.2bp (23.4bp)	PRDM1/MA0508.3/Jaspar(0.708) More Information | Similar Motifs Found	motif file (matrix)
7	TAGGACCTGGAA	1e-43	-9.966e+01	0.20%	0.01%	481.7bp (24.1bp)	PU.1:IRF8(ETS:IRF)/pDC-Irf8-ChIP-Seq(GSE66899)/Homer(0.742) More Information | Similar Motifs Found	motif file (matrix)
8	ACTGTAGGAC	1e-31	-7.231e+01	0.18%	0.01%	438.2bp (13.4bp)	ZBTB32/MA1580.1/Jaspar(0.636) More Information | Similar Motifs Found	motif file (matrix)
9	CCTCGAGAAA	1e-30	-7.017e+01	0.22%	0.02%	432.5bp (22.4bp)	ZBTB26/MA1579.1/Jaspar(0.725) More Information | Similar Motifs Found	motif file (matrix)
10	TCCGCTCTGTTG	1e-28	-6.487e+01	0.12%	0.01%	61.0bp (28.3bp)	ZNF768(Zf)/Rajj-ZNF768-ChIP-Seq(GSE111879)/Homer(0.693) More Information | Similar Motifs Found	motif file (matrix)
11	CTGTGCCCTC	1e-26	-6.016e+01	5.46%	3.92%	280.7bp (31.3bp)	HIC2/MA0738.1/Jaspar(0.705) More Information | Similar Motifs Found	motif file (matrix)
12	ACAGGCCAGGGT	1e-24	-5.580e+01	4.10%	2.82%	448.4bp (31.1bp)	Zfx/MA0146.2/Jaspar(0.729) More Information | Similar Motifs Found	motif file (matrix)
13	GTGGACTCTTGT	1e-23	-5.428e+01	0.10%	0.00%	294.1bp (0.0bp)	PB0134.1_Hnf4a_2/Jaspar(0.695) More Information | Similar Motifs Found	motif file (matrix)
14	GTGGGTCCCCCA	1e-22	-5.171e+01	1.83%	1.05%	358.3bp (31.1bp)	ZNF692(Zf)/HEK293-ZNF692.GFP-ChIP-Seq(GSE58341)/Homer(0.738) More Information | Similar Motifs Found	motif file (matrix)
15	GCTATGGAAACG	1e-22	-5.084e+01	0.10%	0.01%	44.2bp (43.6bp)	NFAT5/MA0606.1/Jaspar(0.716) More Information | Similar Motifs Found	motif file (matrix)
16	CGATGTGGTT	1e-21	-4.888e+01	0.12%	0.01%	124.9bp (35.2bp)	RUNX-AML(Runt)/CD4+-PolII-ChIP-Seq(Barski_et_al.)/Homer(0.795) More Information | Similar Motifs Found	motif file (matrix)
17	GCCATTTTCGAT	1e-20	-4.770e+01	0.63%	0.24%	576.6bp (29.0bp)	YY1/MA0095.2/Jaspar(0.705) More Information | Similar Motifs Found	motif file (matrix)
18	TAAACTGAGAGC	1e-20	-4.748e+01	0.15%	0.02%	98.7bp (25.4bp)	SIX1/MA1118.1/Jaspar(0.665) More Information | Similar Motifs Found	motif file (matrix)
19	ATGGACAGAC	1e-20	-4.745e+01	0.09%	0.01%	64.2bp (7.9bp)	Bcl11a(Zf)/HSPC-BCL11A-ChIP-Seq(GSE104676)/Homer(0.649) More Information | Similar Motifs Found	motif file (matrix)
20	ATGGAATGGAAT	1e-20	-4.745e+01	0.09%	0.01%	88.1bp (27.7bp)	TEAD1(TEAD)/HepG2-TEAD1-ChIP-Seq(Encode)/Homer(0.689) More Information | Similar Motifs Found	motif file (matrix)
21	TTTTCTATTC	1e-19	-4.466e+01	0.23%	0.04%	253.5bp (24.1bp)	Stat2/MA1623.1/Jaspar(0.751) More Information | Similar Motifs Found	motif file (matrix)
22	AAAAACGT	1e-19	-4.466e+01	0.23%	0.04%	488.7bp (22.1bp)	PB0131.1_Gmeb1_2/Jaspar(0.657) More Information | Similar Motifs Found	motif file (matrix)
23	CATAGTCAGGGC	1e-19	-4.411e+01	0.09%	0.00%	794.6bp (0.0bp)	ZNF416(Zf)/HEK293-ZNF416.GFP-ChIP-Seq(GSE58341)/Homer(0.594) More Information | Similar Motifs Found	motif file (matrix)
24	GTCACAGTGCTC	1e-18	-4.316e+01	0.11%	0.01%	72.8bp (21.1bp)	PB0099.1_Zfp691_1/Jaspar(0.738) More Information | Similar Motifs Found	motif file (matrix)
25	CGCCGTGTGGTC	1e-18	-4.151e+01	0.37%	0.11%	342.8bp (29.0bp)	RUNX2(Runt)/PCa-RUNX2-ChIP-Seq(GSE33889)/Homer(0.696) More Information | Similar Motifs Found	motif file (matrix)
26	CCAATCTGCCCA	1e-16	-3.883e+01	1.18%	0.64%	253.4bp (31.0bp)	HIC2/MA0738.1/Jaspar(0.668) More Information | Similar Motifs Found	motif file (matrix)
27	AGAGCCCG	1e-16	-3.866e+01	6.23%	4.90%	324.3bp (30.1bp)	PB0133.1_Hic1_2/Jaspar(0.730) More Information | Similar Motifs Found	motif file (matrix)
28	GCTCCGAG	1e-16	-3.737e+01	5.41%	4.19%	263.9bp (30.9bp)	POL013.1_MED-1/Jaspar(0.755) More Information | Similar Motifs Found	motif file (matrix)
29	GTACCCGTTC	1e-15	-3.580e+01	2.53%	1.74%	300.7bp (30.3bp)	PB0156.1_Plagl1_2/Jaspar(0.598) More Information | Similar Motifs Found	motif file (matrix)
30	GCATACGG	1e-15	-3.495e+01	2.85%	2.01%	267.9bp (30.3bp)	PB0044.1_Mtf1_1/Jaspar(0.665) More Information | Similar Motifs Found	motif file (matrix)
31	GAATCCCGCAGA	1e-15	-3.492e+01	0.09%	0.01%	78.0bp (24.1bp)	E2F3(E2F)/MEF-E2F3-ChIP-Seq(GSE71376)/Homer(0.599) More Information | Similar Motifs Found	motif file (matrix)
32	GTACACGCGTGT	1e-14	-3.443e+01	0.07%	0.00%	211.4bp (0.0bp)	MNT(bHLH)/HepG2-MNT-ChIP-Seq(Encode)/Homer(0.672) More Information | Similar Motifs Found	motif file (matrix)
33	GGTCAGCATG	1e-13	-3.136e+01	0.19%	0.04%	594.7bp (34.1bp)	MAFF/MA0495.3/Jaspar(0.853) More Information | Similar Motifs Found	motif file (matrix)
34	ATCAGCCACA	1e-12	-2.968e+01	0.08%	0.01%	288.2bp (11.1bp)	RUNX1/MA0002.2/Jaspar(0.674) More Information | Similar Motifs Found	motif file (matrix)
35	TGGTATCT	1e-12	-2.946e+01	2.64%	1.90%	547.8bp (29.9bp)	PB0126.1_Gata5_2/Jaspar(0.763) More Information | Similar Motifs Found	motif file (matrix)
36	TACACACT	1e-12	-2.867e+01	1.45%	0.93%	396.9bp (31.5bp)	MSANTD3/MA1523.1/Jaspar(0.723) More Information | Similar Motifs Found	motif file (matrix)
37 *	CTTGAGCTGT	1e-11	-2.714e+01	0.08%	0.01%	410.2bp (0.0bp)	ZBTB6/MA1581.1/Jaspar(0.707) More Information | Similar Motifs Found	motif file (matrix)
38 *	AGAGACCTTCGG	1e-11	-2.714e+01	0.08%	0.01%	51.8bp (2.6bp)	PRDM14(Zf)/H1-PRDM14-ChIP-Seq(GSE22767)/Homer(0.665) More Information | Similar Motifs Found	motif file (matrix)
39 *	TGGTGGGAAT	1e-9	-2.225e+01	0.07%	0.01%	99.8bp (22.0bp)	RBPJ/MA1116.1/Jaspar(0.768) More Information | Similar Motifs Found	motif file (matrix)
40 *	CAAGACGA	1e-9	-2.151e+01	0.24%	0.08%	651.8bp (29.8bp)	PB0108.1_Atf1_2/Jaspar(0.684) More Information | Similar Motifs Found	motif file (matrix)
41 *	GTTGGTGC	1e-9	-2.107e+01	0.43%	0.21%	447.5bp (29.4bp)	PB0150.1_Mybl1_2/Jaspar(0.674) More Information | Similar Motifs Found	motif file (matrix)
42 *	TGTGACCCTT	1e-7	-1.731e+01	0.08%	0.02%	44.6bp (5.4bp)	PB0049.1_Nr2f2_1/Jaspar(0.778) More Information | Similar Motifs Found	motif file (matrix)
43 *	AAATTCGA	1e-7	-1.726e+01	0.10%	0.03%	545.5bp (35.4bp)	PB0136.1_IRC900814_2/Jaspar(0.693) More Information | Similar Motifs Found	motif file (matrix)
44 *	CATCGACG	1e-6	-1.543e+01	0.05%	0.01%	77.4bp (20.3bp)	PB0179.1_Sp100_2/Jaspar(0.679) More Information | Similar Motifs Found	motif file (matrix)
45 *	GGCCTTGC	1e-6	-1.542e+01	0.86%	0.58%	435.3bp (30.8bp)	SF1(NR)/H295R-Nr5a1-ChIP-Seq(GSE44220)/Homer(0.805) More Information | Similar Motifs Found	motif file (matrix)
46 *	GCCTTAGG	1e-3	-7.009e+00	13.51%	12.77%	358.1bp (30.7bp)	TFAP2B(var.2)/MA0812.1/Jaspar(0.863) More Information | Similar Motifs Found	motif file (matrix)

Homer *de novo* Motif Results (E14_motif_bg_random/)

Known Motif Enrichment Results
Gene Ontology Enrichment Results
If Homer is having trouble matching a motif to a known motif, try copy/pasting the matrix file into STAMP
More information on motif finding results: HOMER | Description of Results | Tips
Total target sequences = 21154
Total background sequences = 29500
* - possible false positive

Rank	Motif	P-value	log P-pvalue	% of Targets	% of Background	STD(Bg STD)	Best Match/Details	Motif File
1	TCGCCATATT	1e-66	-1.526e+02	0.22%	0.01%	345.7bp (40.3bp)	YY1/MA0095.2/Jaspar(0.734) More Information | Similar Motifs Found	motif file (matrix)
2	CACGTTTTTC	1e-64	-1.484e+02	0.22%	0.00%	289.0bp (0.0bp)	Prdm15/MA1616.1/Jaspar(0.628) More Information | Similar Motifs Found	motif file (matrix)
3	GTCCTACAGTGT	1e-58	-1.348e+02	0.24%	0.01%	305.5bp (7.2bp)	ZKSCAN1(Zf)/HepG2-ZKSCAN1-ChIP-Seq(Encode)/Homer(0.637) More Information | Similar Motifs Found	motif file (matrix)
4	ATTTCTCATTTT	1e-50	-1.155e+02	0.27%	0.02%	600.2bp (24.0bp)	PB0132.1_Hbp1_2/Jaspar(0.660) More Information | Similar Motifs Found	motif file (matrix)
5	AGAGGGATGCAG	1e-43	-1.002e+02	0.19%	0.01%	315.4bp (1.6bp)	PB0124.1_Gabpa_2/Jaspar(0.607) More Information | Similar Motifs Found	motif file (matrix)
6	ATGATTTTCAGT	1e-37	-8.695e+01	0.18%	0.01%	273.0bp (28.2bp)	IRF4(IRF)/GM12878-IRF4-ChIP-Seq(GSE32465)/Homer(0.682) More Information | Similar Motifs Found	motif file (matrix)
7	CCAAGTCCGTGT	1e-25	-5.876e+01	0.15%	0.01%	389.3bp (0.0bp)	HNF4G/MA0484.2/Jaspar(0.615) More Information | Similar Motifs Found	motif file (matrix)
8	CTTAGCATGGGT	1e-24	-5.620e+01	0.10%	0.01%	60.0bp (4.1bp)	Brn1(POU,Homeobox)/NPC-Brn1-ChIP-Seq(GSE35496)/Homer(0.657) More Information | Similar Motifs Found	motif file (matrix)
9	CGAACCTTGT	1e-24	-5.588e+01	0.13%	0.01%	168.2bp (32.2bp)	HINFP/MA0131.2/Jaspar(0.604) More Information | Similar Motifs Found	motif file (matrix)
10	CCAGATGGAACG	1e-24	-5.588e+01	0.13%	0.01%	87.2bp (19.7bp)	NEUROG2(var.2)/MA1642.1/Jaspar(0.796) More Information | Similar Motifs Found	motif file (matrix)
11	GAGTCTGGAA	1e-22	-5.086e+01	5.83%	4.39%	337.6bp (30.2bp)	Hand1::Tcf3/MA0092.1/Jaspar(0.716) More Information | Similar Motifs Found	motif file (matrix)
12	TGGGCGCATT	1e-19	-4.478e+01	0.32%	0.08%	137.5bp (27.6bp)	PB0143.1_Klf7_2/Jaspar(0.677) More Information | Similar Motifs Found	motif file (matrix)
13	CCCGCTGCGT	1e-18	-4.250e+01	6.42%	5.03%	211.6bp (28.5bp)	Zic2(Zf)/ESC-Zic2-ChIP-Seq(SRP197560)/Homer(0.802) More Information | Similar Motifs Found	motif file (matrix)
14	GGTTTCCGTC	1e-17	-4.035e+01	0.27%	0.07%	429.6bp (24.3bp)	PB0035.1_Irf5_1/Jaspar(0.775) More Information | Similar Motifs Found	motif file (matrix)
15	AGTGCTGAGT	1e-17	-3.963e+01	0.08%	0.00%	109.2bp (0.0bp)	Bach1::Mafk/MA0591.1/Jaspar(0.781) More Information | Similar Motifs Found	motif file (matrix)
16	TTTCATCCCCGA	1e-17	-3.963e+01	0.08%	0.01%	329.7bp (27.2bp)	PH0124.1_Obox5_1/Jaspar(0.673) More Information | Similar Motifs Found	motif file (matrix)
17	GAATGGCTTGAG	1e-16	-3.893e+01	0.10%	0.01%	78.9bp (0.5bp)	Srebp1a(bHLH)/HepG2-Srebp1a-ChIP-Seq(GSE31477)/Homer(0.636) More Information | Similar Motifs Found	motif file (matrix)
18	TGTTGGTTGGCA	1e-16	-3.893e+01	0.10%	0.01%	39.1bp (13.5bp)	PB0029.1_Hic1_1/Jaspar(0.795) More Information | Similar Motifs Found	motif file (matrix)
19	TCAGCGGATG	1e-15	-3.633e+01	0.12%	0.02%	49.9bp (24.5bp)	ETS2/MA1484.1/Jaspar(0.674) More Information | Similar Motifs Found	motif file (matrix)
20	CATCTCTG	1e-15	-3.572e+01	4.88%	3.78%	349.7bp (31.2bp)	ZNF768(Zf)/Rajj-ZNF768-ChIP-Seq(GSE111879)/Homer(0.720) More Information | Similar Motifs Found	motif file (matrix)
21	GGGTACCC	1e-15	-3.476e+01	10.30%	8.71%	255.5bp (30.4bp)	PB0156.1_Plagl1_2/Jaspar(0.796) More Information | Similar Motifs Found	motif file (matrix)
22	TAATACAC	1e-14	-3.377e+01	3.31%	2.43%	391.5bp (29.7bp)	Foxh1(Forkhead)/hESC-FOXH1-ChIP-Seq(GSE29422)/Homer(0.765) More Information | Similar Motifs Found	motif file (matrix)
23	GAATGGAATGGA	1e-14	-3.338e+01	0.07%	0.01%	78.1bp (15.4bp)	PB0098.1_Zfp410_1/Jaspar(0.706) More Information | Similar Motifs Found	motif file (matrix)
24	GATTTTCTGAGG	1e-14	-3.338e+01	0.07%	0.00%	124.8bp (0.0bp)	HOXC10/MA0905.1/Jaspar(0.729) More Information | Similar Motifs Found	motif file (matrix)
25	GGTGATTAGGCT	1e-14	-3.338e+01	0.07%	0.01%	43.3bp (5.4bp)	EVX2/MA0888.1/Jaspar(0.675) More Information | Similar Motifs Found	motif file (matrix)
26	CCAGGGGCCAGC	1e-13	-3.148e+01	2.56%	1.83%	223.7bp (31.5bp)	Plagl1/MA1615.1/Jaspar(0.805) More Information | Similar Motifs Found	motif file (matrix)
27	TAGCATGCCCTC	1e-13	-3.110e+01	0.10%	0.01%	47.6bp (17.5bp)	HIC2/MA0738.1/Jaspar(0.641) More Information | Similar Motifs Found	motif file (matrix)
28	GTGAAACGGAAG	1e-13	-3.105e+01	0.09%	0.01%	126.1bp (14.6bp)	ELF5/MA0136.2/Jaspar(0.675) More Information | Similar Motifs Found	motif file (matrix)
29	AGTCATGTGAGC	1e-12	-2.853e+01	0.08%	0.01%	170.7bp (5.3bp)	USF1/MA0093.3/Jaspar(0.804) More Information | Similar Motifs Found	motif file (matrix)
30	GGTCCCATGAAC	1e-12	-2.853e+01	0.08%	0.01%	41.5bp (9.8bp)	MITF(bHLH)/MastCells-MITF-ChIP-Seq(GSE48085)/Homer(0.667) More Information | Similar Motifs Found	motif file (matrix)
31 *	AGTATCGA	1e-11	-2.738e+01	0.06%	0.01%	109.8bp (0.0bp)	PB0036.1_Irf6_1/Jaspar(0.729) More Information | Similar Motifs Found	motif file (matrix)
32 *	CGCATCCT	1e-11	-2.690e+01	14.68%	13.05%	200.0bp (30.4bp)	ETS:RUNX(ETS,Runt)/Jurkat-RUNX1-ChIP-Seq(GSE17954)/Homer(0.693) More Information | Similar Motifs Found	motif file (matrix)
33 *	GCCTTTGAGT	1e-11	-2.606e+01	0.08%	0.01%	89.0bp (7.4bp)	TCF7/MA0769.2/Jaspar(0.711) More Information | Similar Motifs Found	motif file (matrix)
34 *	GGTGTTCGCA	1e-11	-2.575e+01	0.10%	0.02%	73.1bp (19.6bp)	ZBTB7C/MA0695.1/Jaspar(0.673) More Information | Similar Motifs Found	motif file (matrix)
35 *	CTCTGGGC	1e-11	-2.538e+01	8.13%	6.92%	266.3bp (31.2bp)	ZNF416(Zf)/HEK293-ZNF416.GFP-ChIP-Seq(GSE58341)/Homer(0.789) More Information | Similar Motifs Found	motif file (matrix)
36 *	TGCGTATG	1e-10	-2.480e+01	0.90%	0.54%	443.6bp (28.6bp)	NEUROD2/MA0668.1/Jaspar(0.673) More Information | Similar Motifs Found	motif file (matrix)
37 *	TACGAGTC	1e-10	-2.370e+01	1.27%	0.84%	106.9bp (30.5bp)	MNT(bHLH)/HepG2-MNT-ChIP-Seq(Encode)/Homer(0.692) More Information | Similar Motifs Found	motif file (matrix)
38 *	CGACTAGC	1e-9	-2.202e+01	0.34%	0.15%	65.8bp (28.4bp)	Smad4/MA1153.1/Jaspar(0.673) More Information | Similar Motifs Found	motif file (matrix)
39 *	TGGCCTCGCGCA	1e-9	-2.175e+01	0.10%	0.02%	67.6bp (20.0bp)	TCFL5/MA0632.2/Jaspar(0.634) More Information | Similar Motifs Found	motif file (matrix)
40 *	AATAGAGG	1e-9	-2.087e+01	0.53%	0.29%	229.8bp (27.3bp)	PB0128.1_Gcm1_2/Jaspar(0.715) More Information | Similar Motifs Found	motif file (matrix)
41 *	TAGCAGCCTG	1e-8	-2.025e+01	0.08%	0.01%	48.7bp (26.0bp)	Smad3(MAD)/NPC-Smad3-ChIP-Seq(GSE36673)/Homer(0.606) More Information | Similar Motifs Found	motif file (matrix)
42 *	ACTCGACG	1e-6	-1.472e+01	0.05%	0.01%	77.3bp (42.3bp)	PB0179.1_Sp100_2/Jaspar(0.731) More Information | Similar Motifs Found	motif file (matrix)
43 *	CGCACTTA	1e-5	-1.343e+01	0.08%	0.02%	127.1bp (24.3bp)	ISL2/MA0914.1/Jaspar(0.870) More Information | Similar Motifs Found	motif file (matrix)
44 *	GCTCCTCGCT	1e-3	-9.124e+00	0.06%	0.02%	51.0bp (27.4bp)	ZNF263/MA0528.2/Jaspar(0.682) More Information | Similar Motifs Found	motif file (matrix)

Homer *de novo* Motif Results (E16_motif_bg_random/)

Known Motif Enrichment Results
Gene Ontology Enrichment Results
If Homer is having trouble matching a motif to a known motif, try copy/pasting the matrix file into STAMP
More information on motif finding results: HOMER | Description of Results | Tips
Total target sequences = 30909
Total background sequences = 34000
* - possible false positive

Rank	Motif	P-value	log P-pvalue	% of Targets	% of Background	STD(Bg STD)	Best Match/Details	Motif File
1	GGAATATGGCGA	1e-70	-1.615e+02	0.17%	0.00%	342.2bp (0.0bp)	TEAD2/MA1121.1/Jaspar(0.660)<br>More Information | Similar Motifs Found	motif file (matrix)
2	GTTTCTCATTTT	1e-58	-1.357e+02	0.18%	0.01%	355.1bp (19.0bp)	Stat2/MA1623.1/Jaspar(0.640)<br>More Information | Similar Motifs Found	motif file (matrix)
3	CACTATAGGACC	1e-49	-1.151e+02	0.13%	0.00%	305.5bp (0.0bp)	PB0194.1_Zbtb12_2/Jaspar(0.573)<br>More Information | Similar Motifs Found	motif file (matrix)
4	AACCGGACAAAG	1e-47	-1.095e+02	0.15%	0.01%	275.5bp (21.5bp)	PB0166.1_Sox12_2/Jaspar(0.718)<br>More Information | Similar Motifs Found	motif file (matrix)
5	GTTTTTCCGT	1e-46	-1.062e+02	0.22%	0.02%	276.8bp (32.6bp)	NFATC3/MA0625.1/Jaspar(0.713)<br>More Information | Similar Motifs Found	motif file (matrix)
6	TCTTCAGTTTTC	1e-45	-1.040e+02	0.12%	0.01%	253.5bp (0.0bp)	Stat2/MA1623.1/Jaspar(0.801)<br>More Information | Similar Motifs Found	motif file (matrix)
7	ATTTAGAAACCC	1e-43	-1.004e+02	0.12%	0.01%	1112.9bp (23.4bp)	CHR(?)/Hela-CellCycle-Expression/Homer(0.675)<br>More Information | Similar Motifs Found	motif file (matrix)
8	TGTCAGTGATTT	1e-35	-8.165e+01	0.12%	0.01%	2751.7bp (19.7bp)	PB0195.1_Zbtb3_2/Jaspar(0.752)<br>More Information | Similar Motifs Found	motif file (matrix)
9	TTCGAGGGGGCT	1e-32	-7.572e+01	0.12%	0.01%	90.2bp (13.9bp)	CTCFL/MA1102.2/Jaspar(0.716)<br>More Information | Similar Motifs Found	motif file (matrix)
10	GCGCGCTAAT	1e-30	-7.002e+01	3.47%	2.41%	711.1bp (33.9bp)	PB0008.1_E2F2_1/Jaspar(0.704)<br>More Information | Similar Motifs Found	motif file (matrix)
11	GACCCTAAGTGC	1e-30	-6.991e+01	0.11%	0.01%	60.3bp (7.3bp)	ISL2/MA0914.1/Jaspar(0.682)<br>More Information | Similar Motifs Found	motif file (matrix)
12	CCCGAAGG	1e-30	-6.985e+01	17.55%	15.16%	498.4bp (35.2bp)	PB0086.1_Tcfap2b_1/Jaspar(0.740)<br>More Information | Similar Motifs Found	motif file (matrix)
13	TAGCCACACGCA	1e-29	-6.892e+01	0.09%	0.00%	84.7bp (13.1bp)	Klf4(Zf)/mES-Klf4-ChIP-Seq(GSE11431)/Homer(0.768)<br>More Information | Similar Motifs Found	motif file (matrix)
14	GAGTTGATTTGT	1e-28	-6.559e+01	0.09%	0.01%	94.9bp (0.4bp)	PBX2/MA1113.2/Jaspar(0.622)<br>More Information | Similar Motifs Found	motif file (matrix)
15	CGGCTGAGATGA	1e-28	-6.559e+01	0.09%	0.00%	88.4bp (0.0bp)	Zic(Zf)/Cerebellum-ZIC1.2-ChIP-Seq(GSE60731)/Homer(0.606)<br>More Information | Similar Motifs Found	motif file (matrix)
16	GCTCCGAGTCTT	1e-28	-6.559e+01	0.09%	0.00%	170.9bp (0.0bp)	POL013.1_MED-1/Jaspar(0.686)<br>More Information | Similar Motifs Found	motif file (matrix)
17	GATTTGGG	1e-28	-6.448e+01	1.58%	0.93%	898.0bp (35.5bp)	GSC2/MA0891.1/Jaspar(0.667)<br>More Information | Similar Motifs Found	motif file (matrix)
18	TAGGAACGAG	1e-27	-6.420e+01	0.11%	0.01%	84.5bp (14.3bp)	RFX7/MA1554.1/Jaspar(0.653)<br>More Information | Similar Motifs Found	motif file (matrix)
19	CTGGGGCCCAGT	1e-25	-5.896e+01	5.01%	3.82%	667.3bp (32.3bp)	ZNF692(Zf)/HEK293-ZNF692.GFP-ChIP-Seq(GSE58341)/Homer(0.799)<br>More Information | Similar Motifs Found	motif file (matrix)
20	CTTTAGCG	1e-24	-5.682e+01	2.61%	1.78%	987.3bp (36.7bp)	Nkx2-5(var.2)/MA0503.1/Jaspar(0.674)<br>More Information | Similar Motifs Found	motif file (matrix)
21	TTTGCTGTGT	1e-24	-5.674e+01	0.26%	0.07%	617.4bp (31.3bp)	POL009.1_DCE_S_II/Jaspar(0.709)<br>More Information | Similar Motifs Found	motif file (matrix)
22	TCTCGGTGAGCG	1e-24	-5.587e+01	0.10%	0.01%	100.8bp (24.2bp)	PB0140.1_Irf6_2/Jaspar(0.676)<br>More Information | Similar Motifs Found	motif file (matrix)
23	TTTGACTCAG	1e-23	-5.316e+01	0.09%	0.01%	125.6bp (46.9bp)	Bach2(bZIP)/OCILy7-Bach2-ChIP-Seq(GSE44420)/Homer(0.777)<br>More Information | Similar Motifs Found	motif file (matrix)
24	TCGCATCC	1e-22	-5.276e+01	4.41%	3.35%	697.7bp (36.6bp)	TEAD4(TEA)/Tropoblast-Tead4-ChIP-Seq(GSE37350)/Homer(0.638)<br>More Information | Similar Motifs Found	motif file (matrix)
25	TAAGAGTGTC	1e-22	-5.245e+01	0.16%	0.03%	804.3bp (30.6bp)	PB0180.1_Sp4_2/Jaspar(0.630)<br>More Information | Similar Motifs Found	motif file (matrix)
26	GCCGGGTAAA	1e-22	-5.177e+01	7.22%	5.87%	654.1bp (34.6bp)	PB0153.1_Nr2f2_2/Jaspar(0.756)<br>More Information | Similar Motifs Found	motif file (matrix)
27	AAAACGAC	1e-21	-4.988e+01	1.44%	0.88%	1137.2bp (35.2bp)	PH0044.1_Homez/Jaspar(0.723)<br>More Information | Similar Motifs Found	motif file (matrix)
28	CGCTTAGA	1e-21	-4.961e+01	10.66%	9.06%	883.7bp (35.5bp)	BARHL1/MA0877.2/Jaspar(0.586)<br>More Information | Similar Motifs Found	motif file (matrix)
29	AAGGTCTCGG	1e-20	-4.808e+01	5.74%	4.58%	932.3bp (34.0bp)	Esrrb(NR)/mES-Esrrb-ChIP-Seq(GSE11431)/Homer(0.698)<br>More Information | Similar Motifs Found	motif file (matrix)
30	CGAGGTCCGCTG	1e-19	-4.522e+01	0.08%	0.01%	65.5bp (0.0bp)	HINFP/MA0131.2/Jaspar(0.703)<br>More Information | Similar Motifs Found	motif file (matrix)
31	GGGTGCACCCCG	1e-19	-4.522e+01	0.08%	0.01%	82.7bp (10.5bp)	PB0052.1_Plagl1_1/Jaspar(0.659)<br>More Information | Similar Motifs Found	motif file (matrix)
32	TGGCACCGCG	1e-18	-4.358e+01	0.20%	0.05%	366.0bp (39.5bp)	NFIX(var.2)/MA1528.1/Jaspar(0.645)<br>More Information | Similar Motifs Found	motif file (matrix)
33	ATTAGCCATTCC	1e-18	-4.265e+01	0.08%	0.01%	82.0bp (3.8bp)	TEAD3/MA0808.1/Jaspar(0.656)<br>More Information | Similar Motifs Found	motif file (matrix)
34	TCCACTCTAG	1e-18	-4.172e+01	0.14%	0.03%	112.5bp (28.4bp)	NKX2-2/MA1645.1/Jaspar(0.703)<br>More Information | Similar Motifs Found	motif file (matrix)
35	ACTCGGGTGTGC	1e-17	-4.008e+01	0.09%	0.01%	103.9bp (18.0bp)	PB0130.1_Gm397_2/Jaspar(0.683)<br>More Information | Similar Motifs Found	motif file (matrix)
36	CGTTAAGG	1e-16	-3.712e+01	2.52%	1.86%	590.5bp (35.3bp)	BARHL1/MA0877.2/Jaspar(0.715)<br>More Information | Similar Motifs Found	motif file (matrix)
37	TGAGAGAACC	1e-15	-3.496e+01	0.10%	0.02%	114.5bp (38.8bp)	MSANTD3/MA1523.1/Jaspar(0.620)<br>More Information | Similar Motifs Found	motif file (matrix)
38	GAATGGAATGGA	1e-14	-3.449e+01	0.05%	0.00%	71.9bp (28.7bp)	PB0098.1_Zfp410_1/Jaspar(0.710)<br>More Information | Similar Motifs Found	motif file (matrix)
39	TGGCCATGCT	1e-14	-3.434e+01	0.13%	0.03%	1647.8bp (27.9bp)	SF1(NR)/H295R-Nr5a1-ChIP-Seq(GSE44220)/Homer(0.677)<br>More Information | Similar Motifs Found	motif file (matrix)
40	GTGTGTATCT	1e-14	-3.277e+01	0.07%	0.01%	57.1bp (39.9bp)	DMRT3/MA0610.1/Jaspar(0.653)<br>More Information | Similar Motifs Found	motif file (matrix)
41	GACCGTAT	1e-13	-3.083e+01	0.64%	0.36%	763.5bp (32.1bp)	PB0106.1_Arid5a_2/Jaspar(0.685)<br>More Information | Similar Motifs Found	motif file (matrix)
42	CTTGCTGTAG	1e-13	-3.042e+01	0.06%	0.01%	83.8bp (11.2bp)	ETV1/MA0761.2/Jaspar(0.659)<br>More Information | Similar Motifs Found	motif file (matrix)
43	GATATCGC	1e-13	-3.042e+01	0.06%	0.01%	304.0bp (10.6bp)	PB0127.1_Gata6_2/Jaspar(0.829)<br>More Information | Similar Motifs Found	motif file (matrix)
44 *	TCACTCGT	1e-11	-2.733e+01	0.30%	0.13%	343.5bp (37.9bp)	TBX1/MA0805.1/Jaspar(0.658)<br>More Information | Similar Motifs Found	motif file (matrix)
45 *	GAAAAGCC	1e-10	-2.493e+01	0.79%	0.50%	652.6bp (34.6bp)	ZNF341(Zf)/EBV-ZNF341-ChIP-Seq(GSE113194)/Homer(0.801)<br>More Information | Similar Motifs Found	motif file (matrix)
46 *	GCCTCGGCTT	1e-8	-2.011e+01	0.07%	0.02%	49.8bp (1.7bp)	Zfx/MA0146.2/Jaspar(0.677)<br>More Information | Similar Motifs Found	motif file (matrix)
