# Supplementary material for: In vivo transition in chromatin accessibility during differentiation of deep-layer excitatory neurons in the neocortex
Source: Development. 2025 Jun 27;152(13):dev204564. doi: 10.1242/dev.204564 (PMC12268177; doi:10.1242/dev.204564)
Supplement: Table S5. [file develop-152-204564-TableS5.zip › TableS5.pdf]

Homer *de novo* Motif Results (E12_unique_motif_bg_random/)

Known Motif Enrichment Results
Gene Ontology Enrichment Results
If Homer is having trouble matching a motif to a known motif, try copy/pasting the matrix file into STAMP
More information on motif finding results: HOMER | Description of Results | Tips
Total target sequences = 21533
Total background sequences = 28611
* - possible false positive

Rank	Motif	P-value	log P-pvalue	% of Targets	% of Background	STD(Bg STD)	Best Match/Details	Motif File
1		1e-38	-8.972e+01	17.16%	13.96%	89.8bp (34.6bp)	PLAGL2/MA1548.1/Jaspar(0.885) More Information | Similar Motifs Found	motif file (matrix)
2		1e-36	-8.513e+01	11.60%	9.01%	91.7bp (34.4bp)	ZFX(Zf)/mES-Zfx-ChIP-Seq(GSE11431)/Homer(0.874) More Information | Similar Motifs Found	motif file (matrix)
3		1e-36	-8.489e+01	11.01%	8.48%	94.0bp (34.0bp)	AR-halfsite(NR)/LNCaP-AR-ChIP-Seq(GSE27824)/Homer(0.664) More Information | Similar Motifs Found	motif file (matrix)
4		1e-34	-7.907e+01	14.59%	11.80%	87.1bp (34.4bp)	EKLF(Zf)/Erythrocyte-Klf1-ChIP-Seq(GSE20478)/Homer(0.710) More Information | Similar Motifs Found	motif file (matrix)
5		1e-29	-6.820e+01	2.04%	1.13%	86.4bp (35.0bp)	FXR(NR),IR1/Liver-FXR-ChIP-Seq(Chong_et_al.)/Homer(0.776) More Information | Similar Motifs Found	motif file (matrix)
6		1e-27	-6.386e+01	0.14%	0.01%	56.2bp (9.5bp)	OSR2/MA1646.1/Jaspar(0.689) More Information | Similar Motifs Found	motif file (matrix)
7		1e-26	-5.996e+01	15.60%	13.10%	85.0bp (34.7bp)	ZKSCAN5/MA1652.1/Jaspar(0.703) More Information | Similar Motifs Found	motif file (matrix)
8		1e-25	-5.791e+01	1.49%	0.78%	80.1bp (32.5bp)	ZBTB6/MA1581.1/Jaspar(0.726) More Information | Similar Motifs Found	motif file (matrix)
9		1e-25	-5.790e+01	0.13%	0.01%	59.9bp (33.5bp)	SPDEF(ETS)/VCaP-SPDEF-ChIP-Seq(SRA014231)/Homer(0.637) More Information | Similar Motifs Found	motif file (matrix)
10		1e-24	-5.650e+01	0.31%	0.06%	60.6bp (35.8bp)	BMYB(HTH)/Hela-BMYB-ChIP-Seq(GSE27030)/Homer(0.661) More Information | Similar Motifs Found	motif file (matrix)
11		1e-24	-5.629e+01	0.18%	0.02%	105.3bp (30.9bp)	Sox3/MA0514.1/Jaspar(0.773) More Information | Similar Motifs Found	motif file (matrix)
12		1e-22	-5.242e+01	0.14%	0.01%	169.2bp (13.0bp)	TBX5/MA0807.1/Jaspar(0.743) More Information | Similar Motifs Found	motif file (matrix)
13		1e-21	-4.874e+01	0.09%	0.01%	71.2bp (9.9bp)	SREBF2/MA0596.1/Jaspar(0.622) More Information | Similar Motifs Found	motif file (matrix)
14		1e-21	-4.836e+01	4.94%	3.66%	86.0bp (36.2bp)	SF1(NR)/H295R-Nr5a1-ChIP-Seq(GSE44220)/Homer(0.881) More Information | Similar Motifs Found	motif file (matrix)
15		1e-20	-4.826e+01	0.20%	0.03%	73.3bp (39.2bp)	ZNF519(Zf)/HEK293-ZNF519.GFP-ChIP-Seq(GSE58341)/Homer(0.633) More Information | Similar Motifs Found	motif file (matrix)
16		1e-20	-4.727e+01	0.13%	0.01%	69.6bp (15.8bp)	PB0110.1_Bcl6b_2/Jaspar(0.685) More Information | Similar Motifs Found	motif file (matrix)
17		1e-20	-4.642e+01	0.11%	0.01%	95.4bp (20.6bp)	RHOXF1/MA0719.1/Jaspar(0.684) More Information | Similar Motifs Found	motif file (matrix)
18		1e-19	-4.546e+01	0.09%	0.01%	52.8bp (12.1bp)	PH0111.1_Nkx2-2/Jaspar(0.732) More Information | Similar Motifs Found	motif file (matrix)
19		1e-18	-4.365e+01	0.11%	0.01%	66.1bp (38.2bp)	OSR1/MA1542.1/Jaspar(0.708) More Information | Similar Motifs Found	motif file (matrix)
20		1e-18	-4.298e+01	0.17%	0.03%	80.1bp (35.3bp)	ETV5/MA0765.2/Jaspar(0.744) More Information | Similar Motifs Found	motif file (matrix)
21		1e-17	-4.092e+01	0.10%	0.01%	95.8bp (31.2bp)	ZNF354C/MA0130.1/Jaspar(0.706) More Information | Similar Motifs Found	motif file (matrix)
22		1e-17	-4.092e+01	0.10%	0.01%	97.9bp (14.3bp)	ZNF682/MA1599.1/Jaspar(0.690) More Information | Similar Motifs Found	motif file (matrix)
23		1e-17	-4.092e+01	0.10%	0.01%	119.8bp (11.7bp)	YY2/MA0748.2/Jaspar(0.662) More Information | Similar Motifs Found	motif file (matrix)
24		1e-17	-3.919e+01	0.22%	0.05%	77.4bp (28.7bp)	TEAD4(TEA)/Tropoblast-Tead4-ChIP-Seq(GSE37350)/Homer(0.653) More Information | Similar Motifs Found	motif file (matrix)
25		1e-16	-3.853e+01	4.33%	3.26%	96.4bp (32.0bp)	PB0075.1_Sp100_1/Jaspar(0.676) More Information | Similar Motifs Found	motif file (matrix)
26		1e-16	-3.823e+01	0.10%	0.01%	61.8bp (29.3bp)	Mef2a(MADS)/HL1-Mef2a.biotin-ChIP-Seq(GSE21529)/Homer(0.799) More Information | Similar Motifs Found	motif file (matrix)
27		1e-16	-3.823e+01	0.10%	0.01%	48.9bp (0.0bp)	PB0153.1_Nr2f2_2/Jaspar(0.675) More Information | Similar Motifs Found	motif file (matrix)
28		1e-16	-3.823e+01	0.10%	0.01%	82.1bp (32.2bp)	ZNF263/MA0528.2/Jaspar(0.840) More Information | Similar Motifs Found	motif file (matrix)
29		1e-16	-3.823e+01	0.10%	0.01%	54.3bp (7.6bp)	ZBTB18/MA0698.1/Jaspar(0.614) More Information | Similar Motifs Found	motif file (matrix)
30		1e-16	-3.770e+01	0.13%	0.02%	52.7bp (11.2bp)	MYB/MA0100.3/Jaspar(0.731) More Information | Similar Motifs Found	motif file (matrix)
31		1e-15	-3.559e+01	0.09%	0.01%	96.1bp (0.0bp)	ZNF263/MA0528.2/Jaspar(0.635) More Information | Similar Motifs Found	motif file (matrix)
32		1e-15	-3.559e+01	0.09%	0.01%	74.5bp (26.5bp)	PB0155.1_Osr2_2/Jaspar(0.668) More Information | Similar Motifs Found	motif file (matrix)
33		1e-14	-3.446e+01	7.38%	6.05%	89.2bp (34.7bp)	RHOXF1/MA0719.1/Jaspar(0.715) More Information | Similar Motifs Found	motif file (matrix)
34		1e-14	-3.300e+01	0.09%	0.01%	105.5bp (8.3bp)	Foxf1(Forkhead)/Lung-Foxf1-ChIP-Seq(GSE77951)/Homer(0.713) More Information | Similar Motifs Found	motif file (matrix)
35		1e-14	-3.271e+01	0.10%	0.01%	69.3bp (16.4bp)	Znf281/MA1630.1/Jaspar(0.607) More Information | Similar Motifs Found	motif file (matrix)
36		1e-13	-3.122e+01	0.11%	0.02%	60.4bp (29.7bp)	ZNF354C/MA0130.1/Jaspar(0.706) More Information | Similar Motifs Found	motif file (matrix)
37		1e-13	-3.082e+01	0.91%	0.51%	88.0bp (28.5bp)	POL010.1_DCE_S_III/Jaspar(0.676) More Information | Similar Motifs Found	motif file (matrix)
38		1e-12	-2.987e+01	0.07%	0.01%	56.3bp (17.0bp)	Tbx6(T-box)/ESC-Tbx6-ChIP-Seq(GSE93524)/Homer(0.686) More Information | Similar Motifs Found	motif file (matrix)
39		1e-12	-2.930e+01	0.32%	0.11%	75.5bp (31.6bp)	PAX5/MA0014.3/Jaspar(0.718) More Information | Similar Motifs Found	motif file (matrix)
40		1e-12	-2.903e+01	0.36%	0.14%	81.6bp (28.8bp)	NFIA/MA0670.1/Jaspar(0.642) More Information | Similar Motifs Found	motif file (matrix)
41		1e-12	-2.819e+01	0.09%	0.01%	64.4bp (22.5bp)	Bach1::Mafk/MA0591.1/Jaspar(0.877) More Information | Similar Motifs Found	motif file (matrix)
42		1e-12	-2.797e+01	0.08%	0.01%	68.2bp (17.0bp)	NKX2-3/MA0672.1/Jaspar(0.613) More Information | Similar Motifs Found	motif file (matrix)
43 *		1e-10	-2.409e+01	0.06%	0.01%	61.5bp (0.0bp)	SOX14/MA1562.1/Jaspar(0.836) More Information | Similar Motifs Found	motif file (matrix)
44 *		1e-10	-2.317e+01	0.07%	0.01%	71.5bp (14.4bp)	EWS:FLI1-fusion(ETS)/SK_N_MC-EWS:FLI1-ChIP-Seq(SRA014231)/Homer(0.766) More Information | Similar Motifs Found	motif file (matrix)
45 *		1e-9	-2.209e+01	0.56%	0.30%	63.3bp (36.6bp)	NKX2-8/MA0673.1/Jaspar(0.791) More Information | Similar Motifs Found	motif file (matrix)
46 *		1e-8	-1.976e+01	0.07%	0.01%	145.6bp (39.4bp)	NFYA/MA0060.3/Jaspar(0.745) More Information | Similar Motifs Found	motif file (matrix)
47 *		1e-8	-1.862e+01	0.26%	0.11%	100.0bp (24.1bp)	Sox4(HMG)/proB-Sox4-ChIP-Seq(GSE50066)/Homer(0.669) More Information | Similar Motifs Found	motif file (matrix)
48 *		1e-7	-1.841e+01	0.40%	0.21%	64.5bp (32.3bp)	ETV4/MA0764.2/Jaspar(0.653) More Information | Similar Motifs Found	motif file (matrix)
49 *		1e-7	-1.790e+01	0.48%	0.26%	105.8bp (32.9bp)	NKX2-2/MA1645.1/Jaspar(0.988) More Information | Similar Motifs Found	motif file (matrix)
50 *		1e-7	-1.666e+01	0.48%	0.27%	97.4bp (32.3bp)	PB0208.1_Zscan4_2/Jaspar(0.685) More Information | Similar Motifs Found	motif file (matrix)
51 *		1e-6	-1.524e+01	0.14%	0.05%	67.1bp (31.9bp)	E2F7(E2F)/Hela-E2F7-ChIP-Seq(GSE32673)/Homer(0.780) More Information | Similar Motifs Found	motif file (matrix)
52 *		1e-6	-1.488e+01	0.15%	0.05%	66.2bp (24.5bp)	PB0161.1_Rxra_2/Jaspar(0.680) More Information | Similar Motifs Found	motif file (matrix)
53 *		1e-5	-1.279e+01	0.14%	0.05%	78.6bp (35.8bp)	PB0141.1_Isgf3g_2/Jaspar(0.655) More Information | Similar Motifs Found	motif file (matrix)
54 *		1e-3	-8.946e+00	6.16%	5.58%	81.2bp (31.1bp)	ZNF384/MA1125.1/Jaspar(0.934) More Information | Similar Motifs Found	motif file (matrix)

Homer *de novo* Motif Results (E13_unique_motif_bg_random/)

Known Motif Enrichment Results
Gene Ontology Enrichment Results
If Homer is having trouble matching a motif to a known motif, try copy/pasting the matrix file into STAMP
More information on motif finding results: HOMER | Description of Results | Tips
Total target sequences = 17884
Total background sequences = 32283
* - possible false positive

Rank	Motif	P-value	log P-pvalue	% of Targets	% of Background	STD(Bg STD)	Best Match/Details	Motif File
1	AGTGTCCG	1e-33	-7.803e+01	17.66%	14.36%	58.5bp (24.1bp)	HINFP/MA0131.2/Jaspar(0.652) More Information | Similar Motifs Found	motif file (matrix)
2	CTGGGCCCAGGG	1e-33	-7.615e+01	20.12%	16.67%	60.9bp (23.4bp)	Plagl1/MA1615.1/Jaspar(0.772) More Information | Similar Motifs Found	motif file (matrix)
3	CTCACTCAAG	1e-31	-7.163e+01	8.40%	6.19%	57.5bp (23.2bp)	NKX2-5/MA0063.2/Jaspar(0.649) More Information | Similar Motifs Found	motif file (matrix)
4	CGTCCCCAAAGA	1e-26	-6.105e+01	0.17%	0.01%	43.5bp (18.7bp)	EBF1/MA0154.4/Jaspar(0.707) More Information | Similar Motifs Found	motif file (matrix)
5	GGTGACCT	1e-22	-5.251e+01	16.14%	13.53%	58.6bp (24.5bp)	NR4A2/MA0160.1/Jaspar(0.778) More Information | Similar Motifs Found	motif file (matrix)
6	GTCCCCTGTCCA	1e-21	-5.033e+01	0.13%	0.01%	65.8bp (0.0bp)	PB0200.1_Zfp187_2/Jaspar(0.615) More Information | Similar Motifs Found	motif file (matrix)
7	TCCAGGCACA	1e-21	-4.850e+01	0.20%	0.02%	54.8bp (22.8bp)	AR-halfsite(NR)/LNCaP-AR-ChIP-Seq(GSE27824)/Homer(0.788) More Information | Similar Motifs Found	motif file (matrix)
8	CAAATGAGGTGC	1e-20	-4.756e+01	0.10%	0.00%	35.6bp (0.0bp)	RUNX3/MA0684.2/Jaspar(0.647) More Information | Similar Motifs Found	motif file (matrix)
9	GACATAGCAGGC	1e-20	-4.756e+01	0.10%	0.00%	49.9bp (5.7bp)	Zic2/MA1629.1/Jaspar(0.694) More Information | Similar Motifs Found	motif file (matrix)
10	TGTCCTTAAGGT	1e-20	-4.692e+01	0.14%	0.01%	39.2bp (23.4bp)	SF1(NR)/H295R-Nr5a1-ChIP-Seq(GSE44220)/Homer(0.683) More Information | Similar Motifs Found	motif file (matrix)
11	CACTCCATTCCA	1e-17	-4.065e+01	0.09%	0.01%	47.9bp (12.2bp)	PB0091.1_Zbtb3_1/Jaspar(0.695) More Information | Similar Motifs Found	motif file (matrix)
12	GCTCATTTGACC	1e-17	-4.065e+01	0.09%	0.00%	65.6bp (0.0bp)	POU6F2/MA0793.1/Jaspar(0.613) More Information | Similar Motifs Found	motif file (matrix)
13	TACCATCAGCAT	1e-17	-3.974e+01	0.15%	0.02%	33.7bp (6.4bp)	Mafb/MA0117.2/Jaspar(0.669) More Information | Similar Motifs Found	motif file (matrix)
14	TATCTTGATCTC	1e-16	-3.890e+01	0.12%	0.01%	58.8bp (13.1bp)	GATA3(Zf),DR4/iTreg-Gata3-ChIP-Seq(GSE20898)/Homer(0.606) More Information | Similar Motifs Found	motif file (matrix)
15	TTATGAGAGT	1e-16	-3.783e+01	0.13%	0.02%	48.6bp (24.6bp)	POU6F1(var.2)/MA1549.1/Jaspar(0.597) More Information | Similar Motifs Found	motif file (matrix)
16	CCGATTCGGAGG	1e-16	-3.729e+01	0.08%	0.00%	26.9bp (16.5bp)	ZBTB7B/MA0694.1/Jaspar(0.600) More Information | Similar Motifs Found	motif file (matrix)
17	AACCCATTGTCT	1e-15	-3.631e+01	0.12%	0.01%	44.4bp (20.7bp)	Sox6/MA0515.1/Jaspar(0.713) More Information | Similar Motifs Found	motif file (matrix)
18	CTAAAAGTGCAG	1e-15	-3.631e+01	0.12%	0.01%	28.9bp (17.7bp)	PB0146.1_Mafk_2/Jaspar(0.641) More Information | Similar Motifs Found	motif file (matrix)
19	ATGGAAGACG	1e-14	-3.395e+01	0.18%	0.03%	51.0bp (21.1bp)	ZNF263/MA0528.2/Jaspar(0.634) More Information | Similar Motifs Found	motif file (matrix)
20	TCTCCCGTAG	1e-14	-3.351e+01	0.25%	0.06%	55.4bp (25.6bp)	HIF2a(bHLH)/785_O-HIF2a-ChIP-Seq(GSE34871)/Homer(0.628) More Information | Similar Motifs Found	motif file (matrix)
21	CCATCACGCA	1e-14	-3.311e+01	0.41%	0.14%	53.0bp (21.5bp)	Ahr::Arnt/MA0006.1/Jaspar(0.713) More Information | Similar Motifs Found	motif file (matrix)
22	ATCCGGTGGCAG	1e-14	-3.282e+01	0.10%	0.01%	35.1bp (17.6bp)	HIC1(Zf)/Treg-ZBTB29-ChIP-Seq(GSE99889)/Homer(0.696) More Information | Similar Motifs Found	motif file (matrix)
23	TGGTCAATGCCC	1e-13	-3.075e+01	0.07%	0.00%	58.6bp (0.0bp)	FXR(NR),IR1/Liver-FXR-ChIP-Seq(Chong_et_al.)/Homer(0.762) More Information | Similar Motifs Found	motif file (matrix)
24	TTCAACGCGT	1e-13	-3.049e+01	0.16%	0.03%	50.4bp (23.2bp)	SCRT1/MA0743.2/Jaspar(0.631) More Information | Similar Motifs Found	motif file (matrix)
25	GAAGACTCTAAA	1e-13	-3.008e+01	0.09%	0.01%	60.2bp (14.0bp)	PRDM14(Zf)/H1-PRDM14-ChIP-Seq(GSE22767)/Homer(0.590) More Information | Similar Motifs Found	motif file (matrix)
26 *	GAGGCAAACTTC	1e-11	-2.759e+01	0.07%	0.01%	36.3bp (2.7bp)	Zac1(Zf)/Neuro2A-Plagl1-ChIP-Seq(GSE75942)/Homer(0.573) More Information | Similar Motifs Found	motif file (matrix)
27 *	GCAACCATAG	1e-11	-2.741e+01	0.08%	0.01%	43.1bp (26.0bp)	RUNX(Runt)/HPC7-Runx1-ChIP-Seq(GSE22178)/Homer(0.683) More Information | Similar Motifs Found	motif file (matrix)
28 *	CCTATCATCTTG	1e-11	-2.741e+01	0.08%	0.01%	62.8bp (2.6bp)	LEF1/MA0768.1/Jaspar(0.652) More Information | Similar Motifs Found	motif file (matrix)
29 *	GGTGTCTC	1e-11	-2.700e+01	1.58%	1.01%	61.3bp (23.8bp)	PB0117.1_Eomes_2/Jaspar(0.723) More Information | Similar Motifs Found	motif file (matrix)
30 *	GTCACGGAAGAG	1e-10	-2.411e+01	0.09%	0.01%	32.5bp (16.1bp)	Pax2/MA0067.1/Jaspar(0.669) More Information | Similar Motifs Found	motif file (matrix)
31 *	AAGTTGGAGG	1e-10	-2.331e+01	0.13%	0.03%	36.6bp (27.0bp)	YY1(Zf)/Promoter/Homer(0.664) More Information | Similar Motifs Found	motif file (matrix)
32 *	AGCCCTAA	1e-9	-2.227e+01	18.65%	16.88%	58.6bp (23.9bp)	ZFX(Zf)/mES-Zfx-ChIP-Seq(GSE11431)/Homer(0.741) More Information | Similar Motifs Found	motif file (matrix)
33 *	GTAGTTGGGACT	1e-9	-2.225e+01	0.07%	0.01%	39.5bp (20.8bp)	Hic1/MA0739.1/Jaspar(0.594) More Information | Similar Motifs Found	motif file (matrix)
34 *	ATGCATCT	1e-9	-2.199e+01	0.25%	0.09%	59.2bp (24.9bp)	Pit1(Homeobox)/GCrat-Pit1-ChIP-Seq(GSE58009)/Homer(0.711) More Information | Similar Motifs Found	motif file (matrix)
35 *	GCCAGTGTGA	1e-9	-2.184e+01	0.08%	0.01%	68.9bp (14.6bp)	PB0195.1_Zbtb3_2/Jaspar(0.659) More Information | Similar Motifs Found	motif file (matrix)
36 *	AAGACAGAGG	1e-9	-2.095e+01	0.16%	0.04%	45.8bp (26.2bp)	Sox3/MA0514.1/Jaspar(0.697) More Information | Similar Motifs Found	motif file (matrix)
37 *	GAGTACTA	1e-8	-1.988e+01	0.81%	0.48%	51.5bp (19.8bp)	PB0152.1_Nkx3-1_2/Jaspar(0.796) More Information | Similar Motifs Found	motif file (matrix)
38 *	TTACTCTTTGAC	1e-8	-1.978e+01	0.07%	0.01%	28.4bp (0.0bp)	ZNF652/MA1657.1/Jaspar(0.657) More Information | Similar Motifs Found	motif file (matrix)
39 *	GGCAGGTGCG	1e-7	-1.749e+01	0.07%	0.01%	54.9bp (35.3bp)	ASCL1(var.2)/MA1631.1/Jaspar(0.902) More Information | Similar Motifs Found	motif file (matrix)
40 *	TCTCAAAGCA	1e-7	-1.739e+01	0.06%	0.01%	46.8bp (17.8bp)	TCF7/MA0769.2/Jaspar(0.709) More Information | Similar Motifs Found	motif file (matrix)
41 *	GATTGATTGGTT	1e-7	-1.739e+01	0.06%	0.01%	68.6bp (12.5bp)	NFYC/MA1644.1/Jaspar(0.792) More Information | Similar Motifs Found	motif file (matrix)
42 *	ACCCTCCCTT	1e-5	-1.187e+01	0.07%	0.02%	44.7bp (15.4bp)	MAZ/MA1522.1/Jaspar(0.714) More Information | Similar Motifs Found	motif file (matrix)

Homer *de novo* Motif Results (E14_unique_motif_bg_random/)

[Known Motif Enrichment Results](#)
[Gene Ontology Enrichment Results](#)
If Homer is having trouble matching a motif to a known motif, try copy/pasting the matrix file into [STAMP](#)
More information on motif finding results: [HOMER](#) | [Description of Results](#) | [Tips](#)
Total target sequences = 18651
Total background sequences = 31579
* - possible false positive

Rank	Motif	P-value	log P-pvalue	% of Targets	% of Background	STD(Bg STD)	Best Match/Details	Motif File
1	GAGTCTGGAT	1e-28	-6.579e+01	6.45%	4.63%	59.3bp (24.2bp)	Hand1::Tcf3/MA0092.1/Jaspar(0.730) More Information | Similar Motifs Found	motif file (matrix)
2	CATCTCTCGT	1e-26	-6.121e+01	0.14%	0.01%	62.3bp (28.4bp)	PB0140.1_Irf6_2/Jaspar(0.622) More Information | Similar Motifs Found	motif file (matrix)
3	GCACACAGTTGG	1e-26	-6.121e+01	0.14%	0.01%	49.4bp (2.7bp)	ZKSCAN1(Zf)/HepG2-ZKSCAN1-ChIP-Seq(Encode)/Homer(0.688) More Information | Similar Motifs Found	motif file (matrix)
4	GGTGCCCA	1e-26	-6.079e+01	18.52%	15.60%	58.8bp (24.1bp)	PB0133.1_Hic1_2/Jaspar(0.861) More Information | Similar Motifs Found	motif file (matrix)
5	CTGGCCCA	1e-26	-6.063e+01	10.38%	8.15%	60.0bp (24.5bp)	Plagl1/MA1615.1/Jaspar(0.803) More Information | Similar Motifs Found	motif file (matrix)
6	CTGGCCTGGGGA	1e-25	-5.871e+01	2.44%	1.44%	54.8bp (22.7bp)	Zfp809(Zf)/ES-Zfp809-ChIP-Seq(GSE70799)/Homer(0.698) More Information | Similar Motifs Found	motif file (matrix)
7	AGAGGGACCCAA	1e-23	-5.498e+01	0.13%	0.01%	47.4bp (31.3bp)	ZNF692(Zf)/HEK293-ZNF692.GFP-ChIP-Seq(GSE58341)/Homer(0.735) More Information | Similar Motifs Found	motif file (matrix)
8	TAGGAGACTC	1e-22	-5.163e+01	6.59%	4.96%	57.6bp (23.2bp)	PB0203.1_Zfp691_2/Jaspar(0.746) More Information | Similar Motifs Found	motif file (matrix)
9	CTTTGAGAAGTT	1e-21	-4.978e+01	0.17%	0.02%	76.7bp (19.0bp)	TCF7/MA0769.2/Jaspar(0.635) More Information | Similar Motifs Found	motif file (matrix)
10	CCTGTGGGCCCC	1e-21	-4.925e+01	8.80%	6.95%	59.8bp (22.6bp)	AP-2gamma(AP2)/MCF7-TFAP2C-ChIP-Seq(GSE21234)/Homer(0.730) More Information | Similar Motifs Found	motif file (matrix)
11	CCGCTGGATA	1e-20	-4.758e+01	4.42%	3.15%	54.9bp (23.1bp)	Tcf21(bHLH)/ArterySmoothMuscle-Tcf21-ChIP-Seq(GSE61369)/Homer(0.697) More Information | Similar Motifs Found	motif file (matrix)
12	CTCTTGGCAA	1e-20	-4.675e+01	0.40%	0.11%	47.6bp (22.5bp)	NFIC/MA0161.2/Jaspar(0.823) More Information | Similar Motifs Found	motif file (matrix)
13	CCTCTACTGAGC	1e-20	-4.644e+01	0.10%	0.00%	66.4bp (0.0bp)	Unknown-ESC-element(?)/mES-Nanog-ChIP-Seq(GSE11724)/Homer(0.633) More Information | Similar Motifs Found	motif file (matrix)
14	GTGGCTTCTCGC	1e-18	-4.302e+01	0.09%	0.00%	48.7bp (21.2bp)	POL008.1_DCE_S_I/Jaspar(0.637) More Information | Similar Motifs Found	motif file (matrix)
15	GCAGTGCG	1e-17	-4.081e+01	4.58%	3.37%	56.2bp (23.5bp)	PB0091.1_Zbtb3_1/Jaspar(0.755) More Information | Similar Motifs Found	motif file (matrix)
16	TTCTGCTAACTT	1e-17	-4.015e+01	0.17%	0.02%	31.7bp (17.1bp)	PB0041.1_Mafb_1/Jaspar(0.635) More Information | Similar Motifs Found	motif file (matrix)
17	AAGGGACATG	1e-17	-4.013e+01	0.11%	0.01%	46.7bp (32.6bp)	PB0049.1_Nr2f2_1/Jaspar(0.630) More Information | Similar Motifs Found	motif file (matrix)
18	CCGTGCAAGC	1e-17	-4.013e+01	0.11%	0.01%	45.1bp (16.2bp)	BCL6/MA0463.2/Jaspar(0.583) More Information | Similar Motifs Found	motif file (matrix)
19	CAGGCTATGGAC	1e-17	-3.965e+01	0.09%	0.01%	47.8bp (9.7bp)	ZNF322(Zf)/HEK293-ZNF322.GFP-ChIP-Seq(GSE58341)/Homer(0.609) More Information | Similar Motifs Found	motif file (matrix)
20	TTGTCTTT	1e-17	-3.922e+01	7.54%	6.00%	56.0bp (24.1bp)	Nr2e1/MA0676.1/Jaspar(0.792) More Information | Similar Motifs Found	motif file (matrix)
21	CATCAGAGGA	1e-16	-3.879e+01	0.13%	0.01%	45.9bp (26.0bp)	PB0084.1_Tcf7l2_1/Jaspar(0.698) More Information | Similar Motifs Found	motif file (matrix)
22	CCCAGAAGTCAT	1e-16	-3.800e+01	0.16%	0.02%	37.4bp (15.0bp)	Nr2e1/MA0676.1/Jaspar(0.719) More Information | Similar Motifs Found	motif file (matrix)
23	TGACTCCAAATG	1e-16	-3.758e+01	0.12%	0.01%	37.3bp (7.5bp)	TWIST1/MA1123.2/Jaspar(0.623) More Information | Similar Motifs Found	motif file (matrix)
24	CCTTCACTTT	1e-16	-3.753e+01	0.19%	0.03%	45.9bp (15.4bp)	IRF1(IRF)/PBMC-IRF1-ChIP-Seq(GSE43036)/Homer(0.759) More Information | Similar Motifs Found	motif file (matrix)
25	TTCCGTGTGA	1e-16	-3.734e+01	2.68%	1.81%	56.0bp (23.4bp)	NPAS2(bHLH)/Liver-NPAS2-ChIP-Seq(GSE39860)/Homer(0.651) More Information | Similar Motifs Found	motif file (matrix)
26	AGCTCACCCA	1e-16	-3.730e+01	0.10%	0.01%	59.4bp (17.7bp)	Gli2(Zf)/GM2-Gli2-ChIP-Chip(GSE112702)/Homer(0.713) More Information | Similar Motifs Found	motif file (matrix)
27	CCAAGGACCA	1e-14	-3.312e+01	0.08%	0.00%	49.3bp (20.8bp)	SF1(NR)/H295R-Nr5a1-ChIP-Seq(GSE44220)/Homer(0.690) More Information | Similar Motifs Found	motif file (matrix)
28	GTACCACTCCTT	1e-14	-3.312e+01	0.08%	0.00%	26.3bp (0.8bp)	PB0114.1_Egr1_2/Jaspar(0.680) More Information | Similar Motifs Found	motif file (matrix)
29	ACGGAGTA	1e-13	-3.210e+01	1.02%	0.56%	56.1bp (23.5bp)	PH0126.1_Obox6/Jaspar(0.608) More Information | Similar Motifs Found	motif file (matrix)
30	CGTTCATA	1e-13	-3.079e+01	0.16%	0.03%	39.4bp (9.3bp)	PB0170.1_Sox17_2/Jaspar(0.658) More Information | Similar Motifs Found	motif file (matrix)
31	CCCCCGGGGTAG	1e-13	-3.015e+01	0.10%	0.01%	66.9bp (8.9bp)	PB0102.1_Zic2_1/Jaspar(0.827) More Information | Similar Motifs Found	motif file (matrix)
32	AATAGAACAGAC	1e-12	-2.912e+01	0.09%	0.01%	33.6bp (14.0bp)	ZNF341/MA1655.1/Jaspar(0.666) More Information | Similar Motifs Found	motif file (matrix)
33	TAGCGGGT	1e-12	-2.797e+01	0.17%	0.04%	46.7bp (21.9bp)	OVOL2/MA1545.1/Jaspar(0.660) More Information | Similar Motifs Found	motif file (matrix)
34	TGCGCTCCTGAT	1e-12	-2.778e+01	0.10%	0.01%	36.8bp (0.0bp)	E2A(bHLH),near_PU.1/Bcell-PU.1-ChIP-Seq(GSE21512)/Homer(0.688) More Information | Similar Motifs Found	motif file (matrix)
35 *	TGGGGTGTTTTA	1e-11	-2.651e+01	0.08%	0.01%	36.0bp (8.6bp)	SREBF2/MA0596.1/Jaspar(0.660) More Information | Similar Motifs Found	motif file (matrix)
36 *	GGATTATG	1e-11	-2.543e+01	0.48%	0.22%	42.6bp (19.8bp)	PITX2/MA1547.1/Jaspar(0.911) More Information | Similar Motifs Found	motif file (matrix)
37 *	AGTAATCG	1e-10	-2.423e+01	0.17%	0.04%	35.7bp (23.7bp)	BARHL1/MA0877.2/Jaspar(0.674) More Information | Similar Motifs Found	motif file (matrix)
38 *	TCTAATCACA	1e-10	-2.315e+01	0.10%	0.01%	44.8bp (11.3bp)	NFYC/MA1644.1/Jaspar(0.713) More Information | Similar Motifs Found	motif file (matrix)
39 *	AGCACAGATATG	1e-9	-2.098e+01	0.08%	0.01%	42.1bp (4.7bp)	ZNF768(Zf)/Rajj-ZNF768-ChIP-Seq(GSE111879)/Homer(0.630) More Information | Similar Motifs Found	motif file (matrix)
40 *	AGTCGACA	1e-9	-2.091e+01	0.05%	0.01%	67.5bp (10.2bp)	FOXH1/MA0479.1/Jaspar(0.655) More Information | Similar Motifs Found	motif file (matrix)
41 *	CATTCGTG	1e-9	-2.078e+01	0.85%	0.50%	54.7bp (24.7bp)	HIF-1a(bHLH)/MCF7-HIF1a-ChIP-Seq(GSE28352)/Homer(0.716) More Information | Similar Motifs Found	motif file (matrix)
42 *	ACTTTCCA	1e-8	-2.045e+01	0.47%	0.23%	56.2bp (24.1bp)	NFAT(RHD)/Jurkat-NFATC1-ChIP-Seq(Jolma_et_al.)/Homer(0.846) More Information | Similar Motifs Found	motif file (matrix)
43 *	ATGACAGCCC	1e-8	-1.969e+01	0.10%	0.02%	34.3bp (24.7bp)	MEIS2/MA0774.1/Jaspar(0.783) More Information | Similar Motifs Found	motif file (matrix)
44 *	AGGCTCCGGG	1e-6	-1.535e+01	0.08%	0.02%	32.3bp (29.6bp)	POL013.1_MED-1/Jaspar(0.753) More Information | Similar Motifs Found	motif file (matrix)
45 *	TAACTATC	1e-4	-1.132e+01	0.10%	0.03%	31.9bp (17.4bp)	HMBOX1/MA0895.1/Jaspar(0.754) More Information | Similar Motifs Found	motif file (matrix)

Homer *de novo* Motif Results (E16_unique_motif_bg_random/)

Known Motif Enrichment Results
Gene Ontology Enrichment Results
If Homer is having trouble matching a motif to a known motif, try copy/pasting the matrix file into STAMP
More information on motif finding results: HOMER | Description of Results | Tips
Total target sequences = 29401
Total background sequences = 29491
* - possible false positive

Rank	Motif	P-value	log P-pvalue	% of Targets	% of Background	STD(Bg STD)	Best Match/Details	Motif File
1	CCTCATCCCAAA	1e-51	-1.189e+02	0.14%	0.01%	65.5bp (11.0bp)	ZNF263/MA0528.2/Jaspar(0.653) More Information | Similar Motifs Found	motif file (matrix)
2	CTTTGCGC	1e-41	-9.620e+01	13.52%	10.96%	80.6bp (28.6bp)	SOX10/MA0442.2/Jaspar(0.653) More Information | Similar Motifs Found	motif file (matrix)
3	CCACTATGCTCT	1e-40	-9.320e+01	0.12%	0.01%	69.2bp (8.6bp)	LEF1/MA0768.1/Jaspar(0.592) More Information | Similar Motifs Found	motif file (matrix)
4	GTGCCCTGGG	1e-40	-9.219e+01	11.25%	8.95%	82.7bp (27.7bp)	EBF1(EBF)/Near-E2A-ChIP-Seq(GSE21512)/Homer(0.843) More Information | Similar Motifs Found	motif file (matrix)
5	GGCAATGCCAAA	1e-38	-8.768e+01	0.14%	0.01%	77.5bp (27.4bp)	NFIA/MA0670.1/Jaspar(0.723) More Information | Similar Motifs Found	motif file (matrix)
6	CCGTATGA	1e-35	-8.127e+01	11.74%	9.54%	84.4bp (29.7bp)	PB0106.1_Arid5a_2/Jaspar(0.675) More Information | Similar Motifs Found	motif file (matrix)
7	TGCGGACA	1e-33	-7.696e+01	3.34%	2.22%	88.3bp (29.5bp)	GCM2/MA0767.1/Jaspar(0.730) More Information | Similar Motifs Found	motif file (matrix)
8	AGCATCTCATTT	1e-32	-7.536e+01	0.17%	0.02%	80.6bp (24.7bp)	POU6F1(var.2)/MA1549.1/Jaspar(0.618) More Information | Similar Motifs Found	motif file (matrix)
9	CGCGGATAGA	1e-31	-7.277e+01	1.33%	0.69%	91.0bp (28.2bp)	HINFP(Zf)/K562-HINFP.eGFP-ChIP-Seq(Encode)/Homer(0.574) More Information | Similar Motifs Found	motif file (matrix)
10	CCTCTATCTGGA	1e-30	-6.991e+01	0.12%	0.01%	72.8bp (19.4bp)	ZBTB26/MA1579.1/Jaspar(0.656) More Information | Similar Motifs Found	motif file (matrix)
11	CTTAGAGGGACA	1e-29	-6.681e+01	0.13%	0.01%	60.7bp (24.8bp)	PKNOX1/MA0782.2/Jaspar(0.593) More Information | Similar Motifs Found	motif file (matrix)
12	CCTTAATC	1e-28	-6.668e+01	8.18%	6.51%	80.9bp (28.3bp)	CRX(Homeobox)/Retina-Crx-ChIP-Seq(GSE20012)/Homer(0.630) More Information | Similar Motifs Found	motif file (matrix)
13	CGAGTGTGAA	1e-28	-6.620e+01	3.24%	2.22%	73.2bp (27.8bp)	Tbx21(T-box)/GM12878-TBX21-ChIP-Seq(Encode)/Homer(0.737) More Information | Similar Motifs Found	motif file (matrix)
14	GAAAGCCGGTTC	1e-28	-6.559e+01	0.09%	0.00%	93.3bp (0.0bp)	TFCP2/MA0145.3/Jaspar(0.671) More Information | Similar Motifs Found	motif file (matrix)
15	GAAGAACTTCAT	1e-27	-6.229e+01	0.09%	0.01%	55.7bp (3.7bp)	PH0116.1_Nkx2-9/Jaspar(0.652) More Information | Similar Motifs Found	motif file (matrix)
16	GACTCTGAA	1e-26	-6.171e+01	8.06%	6.45%	78.4bp (28.3bp)	Nkx2.1(Homeobox)/LungAC-Nkx2.1-ChIP-Seq(GSE43252)/Homer(0.686) More Information | Similar Motifs Found	motif file (matrix)
17	CCCCAAGT	1e-26	-6.022e+01	3.73%	2.67%	83.7bp (29.2bp)	ELK1/MA0028.2/Jaspar(0.648) More Information | Similar Motifs Found	motif file (matrix)
18	GCCAAATTGC	1e-26	-6.000e+01	0.69%	0.29%	74.3bp (30.4bp)	NFIX/MA0671.1/Jaspar(0.692) More Information | Similar Motifs Found	motif file (matrix)
19	ACCCAAACTCTA	1e-25	-5.903e+01	0.09%	0.01%	55.5bp (17.2bp)	IRF6/MA1509.1/Jaspar(0.709) More Information | Similar Motifs Found	motif file (matrix)
20	GTGTCACG	1e-24	-5.668e+01	1.59%	0.95%	81.2bp (27.9bp)	TBX3/MA1566.1/Jaspar(0.744) More Information | Similar Motifs Found	motif file (matrix)
21	TATGAACCCG	1e-24	-5.665e+01	0.46%	0.16%	88.0bp (27.3bp)	VDR/MA0693.2/Jaspar(0.783) More Information | Similar Motifs Found	motif file (matrix)
22	CCACGCCTTGGA	1e-24	-5.587e+01	0.10%	0.01%	49.9bp (4.3bp)	PB0180.1_Sp4_2/Jaspar(0.734) More Information | Similar Motifs Found	motif file (matrix)
23	ATAAAACACTGG	1e-24	-5.580e+01	0.08%	0.01%	62.0bp (28.5bp)	Sox17(HMG)/Endoderm-Sox17-ChIP-Seq(GSE61475)/Homer(0.681) More Information | Similar Motifs Found	motif file (matrix)
24	CCGAGTAACA	1e-24	-5.527e+01	7.09%	5.67%	80.9bp (28.9bp)	Rfx5(HTH)/GM12878-Rfx5-ChIP-Seq(GSE31477)/Homer(0.725) More Information | Similar Motifs Found	motif file (matrix)
25	AGAAATGTCA	1e-23	-5.362e+01	1.24%	0.70%	79.5bp (29.3bp)	NR4A1/MA1112.2/Jaspar(0.782) More Information | Similar Motifs Found	motif file (matrix)
26	AGCTCCAACGCC	1e-20	-4.783e+01	0.09%	0.01%	36.6bp (15.4bp)	ZNF415(Zf)/HEK293-ZNF415.GFP-ChIP-Seq(GSE58341)/Homer(0.675) More Information | Similar Motifs Found	motif file (matrix)
27	TCACCCAGCTAA	1e-20	-4.783e+01	0.09%	0.01%	34.6bp (2.0bp)	MAFK/MA0496.3/Jaspar(0.601) More Information | Similar Motifs Found	motif file (matrix)
28	GATTTGGAGCCT	1e-20	-4.758e+01	0.12%	0.02%	49.7bp (29.2bp)	E2F2/MA0864.2/Jaspar(0.634) More Information | Similar Motifs Found	motif file (matrix)
29	AATTAATTCT	1e-20	-4.656e+01	0.89%	0.47%	83.3bp (28.8bp)	Lhx3/MA0135.1/Jaspar(0.848) More Information | Similar Motifs Found	motif file (matrix)
30	CGCTGGCCCT	1e-20	-4.652e+01	0.14%	0.02%	81.7bp (32.3bp)	ZNF519(Zf)/HEK293-ZNF519.GFP-ChIP-Seq(GSE58341)/Homer(0.640) More Information | Similar Motifs Found	motif file (matrix)
31	TACGTTTCGA	1e-19	-4.467e+01	0.10%	0.01%	65.3bp (27.5bp)	PB0037.1_Isgf3g_1/Jaspar(0.719) More Information | Similar Motifs Found	motif file (matrix)
32	AGAAGGTCCCTA	1e-18	-4.265e+01	0.09%	0.01%	65.3bp (33.6bp)	PRDM14(Zf)/H1-PRDM14-ChIP-Seq(GSE22767)/Homer(0.696) More Information | Similar Motifs Found	motif file (matrix)
33	TGGGTGTACAAA	1e-18	-4.173e+01	0.15%	0.03%	75.4bp (25.5bp)	PB0104.1_Zscan4_1/Jaspar(0.627) More Information | Similar Motifs Found	motif file (matrix)
34	GTTGCGCT	1e-17	-4.117e+01	1.44%	0.91%	75.1bp (28.1bp)	ZNF449/MA1656.1/Jaspar(0.721) More Information | Similar Motifs Found	motif file (matrix)
35	AATCCTCCTCCG	1e-16	-3.700e+01	0.10%	0.02%	83.6bp (17.3bp)	ZNF263/MA0528.2/Jaspar(0.732) More Information | Similar Motifs Found	motif file (matrix)
36	GGTCACCC	1e-14	-3.423e+01	0.97%	0.59%	81.5bp (28.0bp)	PAX5/MA0014.3/Jaspar(0.795) More Information | Similar Motifs Found	motif file (matrix)
37	TCCTGGAAAG	1e-14	-3.340e+01	0.13%	0.03%	103.7bp (25.9bp)	Bcl6(Zf)/Liver-Bcl6-ChIP-Seq(GSE31578)/Homer(0.851) More Information | Similar Motifs Found	motif file (matrix)
38	TCCTCTCA	1e-13	-3.168e+01	1.59%	1.10%	75.1bp (27.9bp)	Nkx2-5(var.2)/MA0503.1/Jaspar(0.742) More Information | Similar Motifs Found	motif file (matrix)
39	ACTGGTGTGC	1e-13	-3.134e+01	0.08%	0.01%	97.3bp (23.8bp)	PB0208.1_Zscan4_2/Jaspar(0.725) More Information | Similar Motifs Found	motif file (matrix)
40	GCCCAATGCCAT	1e-13	-3.127e+01	0.10%	0.02%	65.7bp (12.5bp)	PB0191.1_Tcfap2c_2/Jaspar(0.678) More Information | Similar Motifs Found	motif file (matrix)
41	CCTCAGAC	1e-13	-3.005e+01	3.50%	2.77%	76.3bp (28.6bp)	TFAP2A/MA0003.4/Jaspar(0.735) More Information | Similar Motifs Found	motif file (matrix)
42	AGCTGCCCCG	1e-12	-2.841e+01	0.11%	0.02%	123.8bp (23.7bp)	Ascl2(bHLH)/ESC-Ascl2-ChIP-Seq(GSE97712)/Homer(0.703) More Information | Similar Motifs Found	motif file (matrix)
43 *	CGATCGTT	1e-11	-2.539e+01	0.08%	0.02%	73.0bp (1.6bp)	SOX9/MA0077.1/Jaspar(0.711) More Information | Similar Motifs Found	motif file (matrix)
44 *	ATTCACTCCTGG	1e-9	-2.134e+01	0.13%	0.04%	68.9bp (14.0bp)	TBX15/MA0803.1/Jaspar(0.694) More Information | Similar Motifs Found	motif file (matrix)
45 *	AGGGATAT	1e-8	-1.993e+01	0.21%	0.09%	57.2bp (21.9bp)	SD0003.1_at_AC_acceptor/Jaspar(0.827) More Information | Similar Motifs Found	motif file (matrix)
46 *	ACCCGCTA	1e-6	-1.584e+01	0.18%	0.08%	71.7bp (32.7bp)	OVOL2/MA1545.1/Jaspar(0.680) More Information | Similar Motifs Found	motif file (matrix)
47 *	GGAAACTGGC	1e-6	-1.492e+01	0.07%	0.02%	51.2bp (24.1bp)	NFATC4/MA1525.1/Jaspar(0.701) More Information | Similar Motifs Found	motif file (matrix)
